# Supplementary material for: The efficacy of single mitochondrial genes at reconciling the complete mitogenome phylogeny—a case study on dwarf chameleons
Source: PeerJ. 2024 Apr 30;12:e17076. doi: 10.7717/peerj.17076 (PMC11067893; doi:10.7717/peerj.17076)
Supplement: Supplemental Information 1 — The efficacy of single mitochondrial genes at reconciling the complete mitogenome phylogeny –A case study on dwarf chameleons [file peerj-12-17076-s001.docx]

**Supplemental Information:**

The efficacy of single mitochondrial genes at reconciling the complete mitogenome phylogeny – A case study on dwarf chameleons

*Published in PeerJ {issue/pages to be added upon publication}*

Devon C. Main^1^, Jody M. Taft^2,3^, Anthony J. Geneva^4^, Bettine Jansen van Vuuren^1^, Krystal A. Tolley^1,2*^

^1^Centre for Ecological Genomics and Wildlife Conservation, University of Johannesburg, Auckland Park, 2006, Johannesburg, South Africa

^2^South African National Biodiversity Institute, Kirstenbosch Research Centre, Private Bag X7, Claremont 7735, South Africa

^3^School of Animal, Plant and Environmental Sciences, University of the Witwatersrand, P.O. Wits, 2050 Johannesburg, South Africa

^4^Department of Biology, Center for Computational and Integrative Biology, Rutgers University-Camden, Camden, NJ, USA

Table of contents

**Figure S1**: A best-supported coalescent phylogeny estimated in ASTRAL---------------------------------2

**Figure S2**: A best-supported maximum likelihood phylogeny showing site concordance factors-------3

**Table S1**: GenBank accession numbers for all sequences generated in this study------------------------ 4


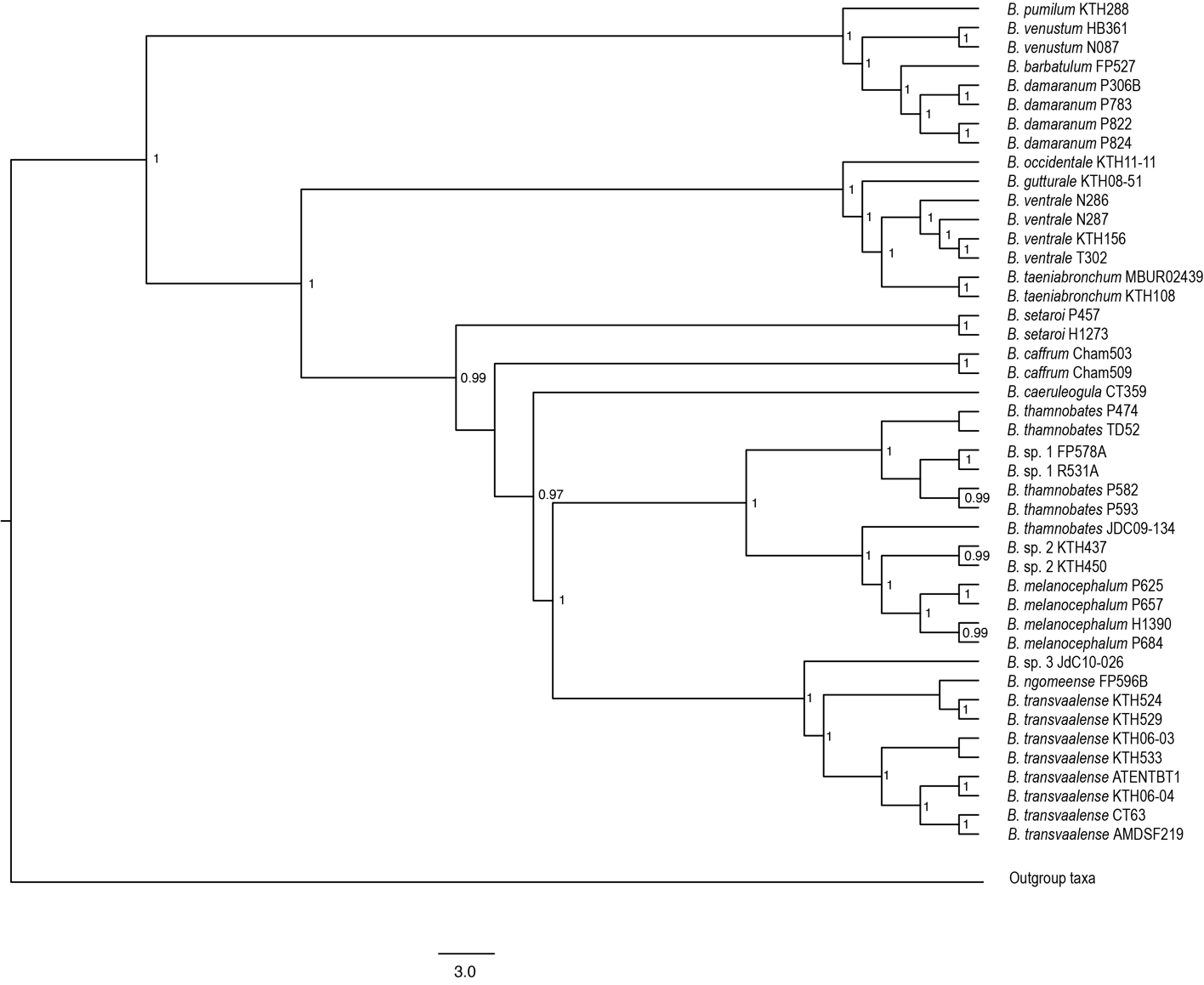


**Figure. S1:** A best-supported coalescent species tree inferred from 15 mitochondrial genes using ASTRAL. Local posterior probability support is indicated on nodes. Support values are only indicated for nodes with PP > 0.95.


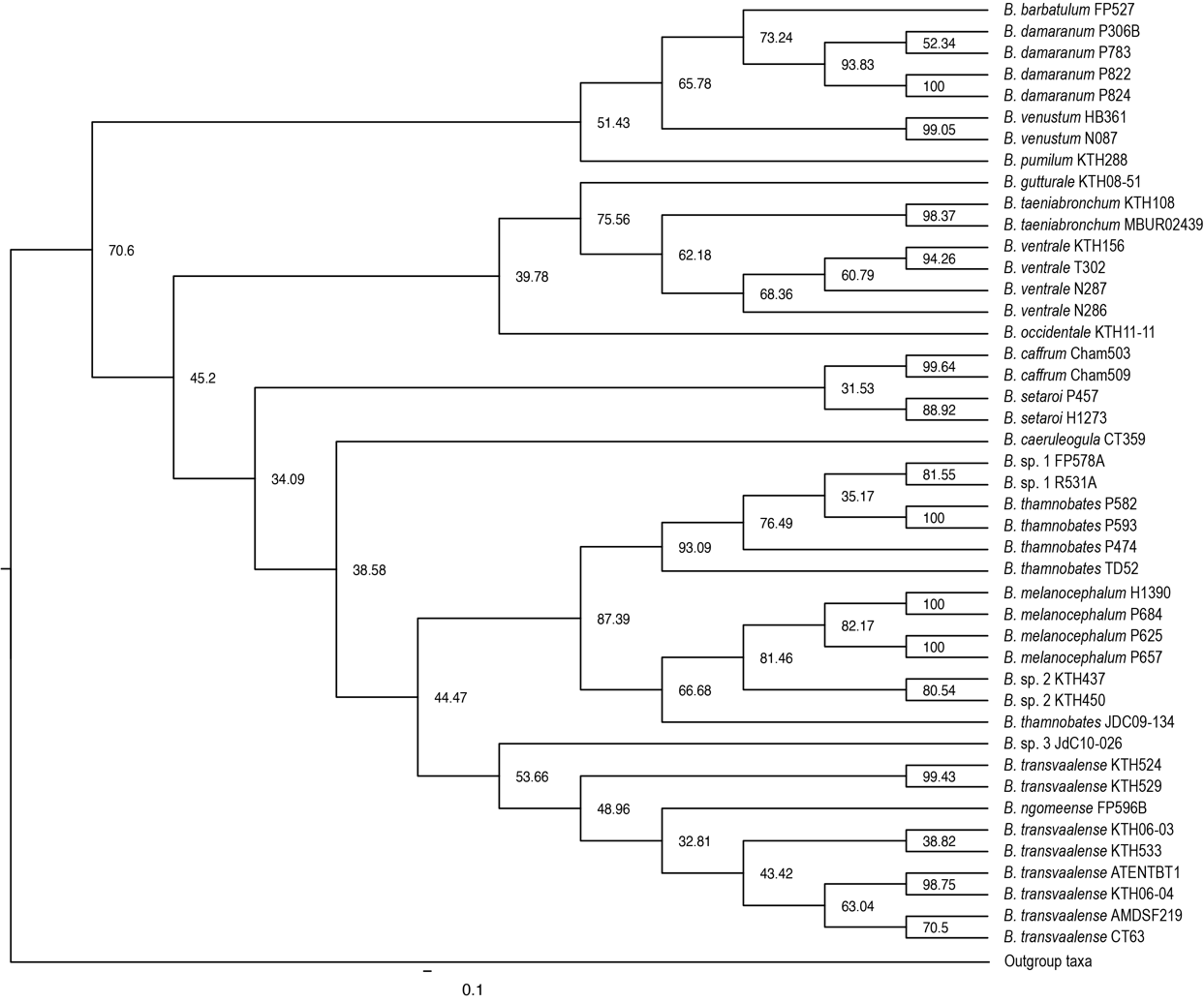


**Figure. S2:** A best-supported maximum likelihood tree inferred from 15 mitochondrial genes using IQ-TREE. Site concordance factors are indicated at the nodes.

**Table S1**: GenBank accession numbers for all Bradypodion sequences published in this study.

| **Species*** | **ID** | ***ATP6*** | ***ATP8*** | ***COI*** | ***COII*** | ***COIII*** | ***CytB*** | ***ND1*** | ***ND2*** | ***ND3*** | ***ND4*** | ***ND4l*** | ***ND5*** | ***ND6*** | ***12S*** | ***16S*** |
| --- | --- | --- | --- | --- | --- | --- | --- | --- | --- | --- | --- | --- | --- | --- | --- | --- |
| *B. barbatulum* | FP527 | OR701174 | OR701218 | OR707264 | OR701042 | OR701086 | OR701130 | OR700866 | OR700778 | OR700822 | OR700910 | OR700998 | OR701262 | OR700954 | OR767167 | OR766795 |
| *B. caeruleogula* | CT359 | OR701173 | OR701217 | OR707263 | OR701041 | OR701085 | OR701129 | OR700865 | OR700777 | OR700821 | OR700909 | OR700997 | OR701261 | OR700953 | OR767166 | OR766794 |
| *B. caffrum* | Cham503 | OR701170 | OR701214 | OR707260 | OR701038 | OR701082 | OR701126 | OR700862 | OR700774 | OR700818 | OR700906 | OR700994 | OR701258 | OR700950 | OR767163 | OR766791 |
| *B. caffrum* | Cham509 | OR701171 | OR701215 | OR707261 | OR701039 | OR701083 | OR701127 | OR700863 | OR700775 | OR700819 | OR700907 | OR700995 | OR701259 | OR700951 | OR767164 | OR766792 |
| *B.* sp. 3 | JdC10-026 | OR701179 | OR701223 | OR707269 | OR701047 | OR701091 | OR701135 | OR700871 | OR700783 | OR700827 | OR700915 | OR701003 | OR701267 | OR700959 | OR767172 | OR766800 |
| *B. damaranum* | P306B | OR701194 | OR701238 | OR707284 | OR701062 | OR701106 | OR701150 | OR700886 | OR700798 | OR700842 | OR700930 | OR701018 | OR701282 | OR700974 | OR767187 | OR766815 |
| *B. damaranum* | P783 | OR701202 | OR701246 | OR707292 | OR701070 | OR701114 | OR701158 | OR700894 | OR700806 | OR700850 | OR700938 | OR701026 | OR701290 | OR700982 | OR767195 | OR766823 |
| *B. damaranum* | P822 | OR701203 | OR701247 | OR707293 | OR701071 | OR701115 | OR701159 | OR700895 | OR700807 | OR700851 | OR700939 | OR701027 | OR701291 | OR700983 | OR767196 | OR766824 |
| *B. damaranum* | P824 | OR701204 | OR701248 | OR707294 | OR701072 | OR701116 | OR701160 | OR700896 | OR700808 | OR700852 | OR700940 | OR701028 | OR701292 | OR700984 | OR767197 | OR766825 |
| *B. gutturale* | KTH08-51 | OR701182 | OR701226 | OR707272 | OR701050 | OR701094 | OR701138 | OR700874 | OR700786 | OR700830 | OR700918 | OR701006 | OR701270 | OR700962 | OR767175 | OR766803 |
| *B. melanocephalum* | H1390 | OR701177 | OR701221 | OR707267 | OR701045 | OR701089 | OR701133 | OR700869 | OR700781 | OR700825 | OR700913 | OR701001 | OR701265 | OR700957 | OR767170 | OR766798 |
| *B. melanocephalum* | P625 | OR701199 | OR701243 | OR707289 | OR701067 | OR701111 | OR701155 | OR700891 | OR700803 | OR700847 | OR700935 | OR701023 | OR701287 | OR700979 | OR767192 | OR766820 |
| *B. melanocephalum* | P657 | OR701200 | OR701244 | OR707290 | OR701068 | OR701112 | OR701156 | OR700892 | OR700804 | OR700848 | OR700936 | OR701024 | OR701288 | OR700980 | OR767193 | OR766821 |
| *B. melanocephalum* | P684 | OR701201 | OR701245 | OR707291 | OR701069 | OR701113 | OR701157 | OR700893 | OR700805 | OR700849 | OR700937 | OR701025 | OR701289 | OR700981 | OR767194 | OR766822 |
| *B. ngomeense* | FP596B | OR701176 | OR701220 | OR707266 | OR701044 | OR701088 | OR701132 | OR700868 | OR700780 | OR700824 | OR700912 | OR701000 | OR701264 | OR700956 | OR767169 | OR766797 |
| *B. occidentale* | KTH11-11 | OR701183 | OR701227 | OR707273 | OR701051 | OR701095 | OR701139 | OR700875 | OR700787 | OR700831 | OR700919 | OR701007 | OR701271 | OR700963 | OR767176 | OR766804 |
| *B. pumilum* | KTH288 | OR701186 | OR701230 | OR707276 | OR701054 | OR701098 | OR701142 | OR700878 | OR700790 | OR700834 | OR700922 | OR701010 | OR701274 | OR700966 | OR767179 | OR766807 |
| *B. setaroi* | P457 | OR701206 | OR701250 | OR707296 | OR701074 | OR701118 | OR701162 | OR700898 | OR700810 | OR700854 | OR700942 | OR701030 | OR701294 | OR700986 | OR767199 | OR766827 |
| *B. setaroi* | H1273 | OR701195 | OR701239 | OR707285 | OR701063 | OR701107 | OR701151 | OR700887 | OR700799 | OR700843 | OR700931 | OR701019 | OR701283 | OR700975 | OR767188 | OR766816 |
| *B.* sp. 1 | FP578A | OR701175 | OR701219 | OR707265 | OR701043 | OR701087 | OR701131 | OR700867 | OR700779 | OR700823 | OR700911 | OR700999 | OR701263 | OR700955 | OR767168 | OR766796 |
| *B.* sp. 1 | R531A | OR701205 | OR701249 | OR707295 | OR701073 | OR701117 | OR701161 | OR700897 | OR700809 | OR700853 | OR700941 | OR701029 | OR701293 | OR700985 | OR767198 | OR766826 |
| *B.* sp. 2 | KTH-437 | OR701187 | OR701231 | OR707277 | OR701055 | OR701099 | OR701143 | OR700879 | OR700791 | OR700835 | OR700923 | OR701011 | OR701275 | OR700967 | OR767180 | OR766808 |
| *B.* sp. 2 | KTH-450 | OR701188 | OR701232 | OR707278 | OR701056 | OR701100 | OR701144 | OR700880 | OR700792 | OR700836 | OR700924 | OR701012 | OR701276 | OR700968 | OR767181 | OR766809 |
| *B. taeniabronchum* | KTH-108 | OR701184 | OR701228 | OR707274 | OR701052 | OR701096 | OR701140 | OR700876 | OR700788 | OR700832 | OR700920 | OR701008 | OR701272 | OR700964 | OR767177 | OR766805 |
| *B. taeniabronchum* | MBUR 02439 | OR701190 | OR701234 | OR707280 | OR701058 | OR701102 | OR701146 | OR700882 | OR700794 | OR700838 | OR700926 | OR701014 | OR701278 | OR700970 | OR767183 | OR766811 |
| *B. thamnobates* | P474 | OR701211 | OR701255 | OR707301 | OR701079 | OR701123 | OR701167 | OR700903 | OR700815 | OR700859 | OR700947 | OR701035 | OR701299 | OR700991 | OR767204 | OR766832 |
| *B. thamnobates* | P582 | OR701196 | OR701240 | OR707286 | OR701064 | OR701108 | OR701152 | OR700888 | OR700800 | OR700844 | OR700932 | OR701020 | OR701284 | OR700976 | OR767189 | OR766817 |
| *B. thamnobates* | P593 | OR701197 | OR701241 | OR707287 | OR701065 | OR701109 | OR701153 | OR700889 | OR700801 | OR700845 | OR700933 | OR701021 | OR701285 | OR700977 | OR767190 | OR766818 |
| *B. thamnobates* | TD52 | OR701198 | OR701242 | OR707288 | OR701066 | OR701110 | OR701154 | OR700890 | OR700802 | OR700846 | OR700934 | OR701022 | OR701286 | OR700978 | OR767191 | OR766819 |
| *B. thamnobates* | JDC09-134 | OR701210 | OR701254 | OR707300 | OR701078 | OR701122 | OR701166 | OR700902 | OR700814 | OR700858 | OR700946 | OR701034 | OR701298 | OR700990 | OR767203 | OR766831 |
| *B. transvaalense* | AMDSF219 | OR701168 | OR701212 | OR707258 | OR701036 | OR701080 | OR701124 | OR700860 | OR700772 | OR700816 | OR700904 | OR700992 | OR701256 | OR700948 | OR767161 | OR766789 |
| *B. transvaalense* | ATENTBT1 | OR701169 | OR701213 | OR707259 | OR701037 | OR701081 | OR701125 | OR700861 | OR700773 | OR700817 | OR700905 | OR700993 | OR701257 | OR700949 | OR767162 | OR766790 |
| *B. transvaalense* | CT-63 | OR701172 | OR701216 | OR707262 | OR701040 | OR701084 | OR701128 | OR700864 | OR700776 | OR700820 | OR700908 | OR700996 | OR701260 | OR700952 | OR767165 | OR766793 |
| *B. transvaalense* | KTH06-03 | OR701180 | OR701224 | OR707270 | OR701048 | OR701092 | OR701136 | OR700872 | OR700784 | OR700828 | OR700916 | OR701004 | OR701268 | OR700960 | OR767173 | OR766801 |
| *B. transvaalense* | KTH06-04 | OR701181 | OR701225 | OR707271 | OR701049 | OR701093 | OR701137 | OR700873 | OR700785 | OR700829 | OR700917 | OR701005 | OR701269 | OR700961 | OR767174 | OR766802 |
| *B. transvaalense* | KTH533 | OR701208 | OR701252 | OR707298 | OR701076 | OR701120 | OR701164 | OR700900 | OR700812 | OR700856 | OR700944 | OR701032 | OR701296 | OR700988 | OR767201 | OR766829 |
| *B. transvaalense* | KTH524 | OR701209 | OR701253 | OR707299 | OR701077 | OR701121 | OR701165 | OR700901 | OR700813 | OR700857 | OR700945 | OR701033 | OR701297 | OR700989 | OR767202 | OR766830 |
| *B. transvaalense* | KTH529 | OR701189 | OR701233 | OR707279 | OR701057 | OR701101 | OR701145 | OR700881 | OR700793 | OR700837 | OR700925 | OR701013 | OR701277 | OR700969 | OR767182 | OR766810 |
| *B. ventrale* | KTH156 | OR701185 | OR701229 | OR707275 | OR701053 | OR701097 | OR701141 | OR700877 | OR700789 | OR700833 | OR700921 | OR701009 | OR701273 | OR700965 | OR767178 | OR766806 |
| *B. ventrale* | N286 | OR701191 | OR701235 | OR707281 | OR701059 | OR701103 | OR701147 | OR700883 | OR700795 | OR700839 | OR700927 | OR701015 | OR701279 | OR700971 | OR767184 | OR766812 |
| *B. ventrale* | N287 | OR701192 | OR701236 | OR707282 | OR701060 | OR701104 | OR701148 | OR700884 | OR700796 | OR700840 | OR700928 | OR701016 | OR701280 | OR700972 | OR767185 | OR766813 |
| *B. ventrale* | T302 | OR701207 | OR701251 | OR707297 | OR701075 | OR701119 | OR701163 | OR700899 | OR700811 | OR700855 | OR700943 | OR701031 | OR701295 | OR700987 | OR767200 | OR766828 |
| *B. venustum* | HB361 | OR701178 | OR701222 | OR707268 | OR701046 | OR701090 | OR701134 | OR700870 | OR700782 | OR700826 | OR700914 | OR701002 | OR701266 | OR700958 | OR767171 | OR766799 |
| *B. venustum* | N087 | OR701193 | OR701237 | OR707283 | OR701061 | OR701105 | OR701149 | OR700885 | OR700797 | OR700841 | OR700929 | OR701017 | OR701281 | OR700973 | OR767186 | OR766814 |

*all species listed are in the genus *Bradypodion*. Sequences can be accessed at https://www.ncbi.nlm.nih.gov/genbank/.
